# Ideal, expected and perceived descriptive norm drunkenness in UK nightlife environments: a cross-sectional study

**DOI:** 10.1186/s12889-019-6802-5

**Published:** 2019-04-27

**Authors:** Karen Hughes, Zara Quigg, Kat Ford, Mark A. Bellis

**Affiliations:** 1 Policy and International Health, Public Health Wales, Clwydian House, Wrexham, LL13 7YP UK; 2 College of Human Sciences, Bangor University, Bangor, LL57 2UW UK; 3 Public Health Institute, Liverpool John Moores University, Liverpool, L2 2QP UK; 4 Bangor Institute for Health and Medical Research, School of Health Sciences, Wrexham, LL13 7YP UK

**Keywords:** Alcohol consumption, Intoxication, Nightlife, Social norms

## Abstract

**Background:**

Drunkenness is common in nightlife environments and studies suggest it can be considered both desirable and normal by nightlife users. We aimed to compare UK nightlife users’ ideal levels of drunkenness to their expected drunkenness on a night out and their perceptions of descriptive nightlife norms.

**Methods:**

A cross-sectional survey with nightlife patrons (*n* = 408, aged 18–35) in three cities. Using a scale from 1 (completely sober) to 10 (very drunk), participants rated: personal drunkenness at survey; expected drunkenness on leaving nightlife; perceived descriptive drunkenness norm in the city’s nightlife; and ideal personal drunkenness. Analyses were limited to those who had or were intending to consume alcohol.

**Results:**

Almost half of participants (46.8%) expected to get drunker than their reported ideal level on the night of survey, rising to four fifths of those with the highest levels of expected drunkenness. 77.9% rated typical nightlife drunkenness ≥8 but only 40.9% expected to reach this level themselves and only 23.1% reported their ideal drunkenness as ≥8. Higher expected drunkenness was associated with higher ideal drunkenness, higher perceived drunkenness norm and later expected home time.

**Conclusions:**

Nightlife users’ perceptions of typical drunkenness in nightlife settings may be elevated and many of the heaviest drinkers are likely to drink beyond their ideal level of drunkenness. Findings can support emerging work to address cultures of intoxication in nightlife environments and suggest that interventions to correct misperceptions of normal levels of nightlife drunkenness may be of benefit.

**Electronic supplementary material:**

The online version of this article (10.1186/s12889-019-6802-5) contains supplementary material, which is available to authorized users.

## Background

Harmful drinking and drunkenness are common features of nightlife environments in many countries [1–6]. As well as damaging the health of drinkers, such drinking behaviours can impose harms on others (e.g. through violence, injury, vandalism and noise [7, 8]) and place major burdens on health and criminal justice systems [9, 10]. In the UK, despite recent trends showing a decline in alcohol use among young people in the general population [11], drunkenness has been a persistent feature of youth nightlife drinking practices for many years, remaining largely unresponsive to campaigns and other initiatives promoting safer drinking [12]. Both here and elsewhere, responses to alcohol problems in nightlife environments have tended to focus on preventing the harms associated with drunkenness (e.g. violence) rather than reducing alcohol use itself [13, 14]. However, there is now a growing recognition of the need to address cultures of intoxication through both controlling alcohol availability [15–18] and challenging social norms that support high levels of drunkenness. Challenging such social norms however requires a better understanding of drinkers’ perceptions regarding drunkenness in nightlife environments.

Alcohol is a central part of socialising for many young people [19] and is reported to be valued for its social functions, such as helping people to ‘loosen up’, increasing confidence, and facilitating bonding and social interaction with peers and potential romantic partners [20, 21]. Studies suggest that young people primarily view intoxication as fun, associated with pleasure, laughter and gregarious behaviour [20, 22, 23]. Stories of drunken exploits on nights out can later provide entertainment and fuel social bonding [24]. In policy and practice, drunkenness is often discussed as a single harmful state, yet in reality it has different meanings to different people. For some, getting drunk may mean achieving the euphoric effects that occur at relatively low blood alcohol concentrations, such as improved mood and increased self-confidence and sociability. For others, it can mean becoming ‘annihilated’ or ‘plastered’ [22]; achieving high levels of blood alcohol concentration that can result in harms such as dizziness, vomiting and unconsciousness. Qualitative studies have found such extreme drunkenness to be a desired goal of alcohol use in some youth cultures, including in the UK [20, 21]. Elsewhere, studies have found young drinkers to hold more negative connotations of drunkenness, reporting a desire to find a threshold at which they are most happy and enacting strategies to maintain this throughout the night (e.g. Italy [25]).

Young people’s personal drinking behaviours are influenced by their perceptions of drinking norms among peers [26, 27]. Thus the more alcohol individuals think other people drink (descriptive norms), the more they tend to drink themselves. Studies routinely identify misperceptions of both descriptive (i.e. typical behaviour) and injunctive (i.e. approval of behaviour) alcohol norms among students [28], with a review concluding that most students believed they drank less and approved of drinking less than their peers [29]. However, despite high levels of drunkenness in nightlife environments, few studies have explored perceptions of drunkenness norms in nightlife patrons. Further, most research exploring the concept of ideal, ‘happy’ levels of drunkenness and adherence to these levels in young drinkers has been qualitative. Here, we present findings from an exploratory study that aimed to gain a self-rated quantitative measure of UK nightlife users’ ideal level of drunkenness, expected level of drunkenness by the end of a night out and perceived descriptive norms regarding levels of nightlife drunkenness. We explore the extent to which individuals exceed their ideal level of drunkenness whilst on a night out and the relationships between ideal and expected drunkenness and perceptions of descriptive nightlife drunkenness norms.

## Methods

A short anonymous questionnaire was developed covering participants’ demographics; nightlife participation (e.g. time out, intended home time); alcohol consumption on the night of survey (converted into standard UK units for analysis, where one unit = 8 mg pure alcohol); whether they had preloaded (i.e. consumed alcohol in a domestic residence before attending licensed premises [30], and asked here as whether they had consumed alcohol before going out, e.g. at home, a friend’s home or a hotel room); and a range of questions on perceptions of drunkenness. These included four questions measured on a scale of 1 to 10, where 1 represented ‘completely sober’ and 10 represented ‘very drunk’: 1) How drunk do you feel at this moment? (referred to as *personal drunkenness at survey*); 2) How drunk do you think you will be when you leave the city centre tonight? (*expected personal drunkenness*); 3) Overall, what do you think is the typical level of drunkenness that people reach on a night out in the city centre? (*perceived city drunkenness norm*); and 4) For you personally, what do you think is the ideal level where you are as happy as you can be after drinking alcohol? (*ideal personal drunkenness*) (see Additional file 1).

We surveyed nightlife users aged 18 and over in the streets of three UK cities during peak nightlife hours (City 1, 10 pm-4.30 am, October 2014; Cities 2 & 3, 9 pm-3 am, January 2015). Researchers approached potential participants, outlined the study verbally and asked if they had time to participate. A study information sheet was provided to those who were interested, and researchers confirmed informed consent prior to proceeding. Thus, consistent with ethics approval, researchers were trained to visually assess potential participants and those assessed as already so heavily intoxicated that they could not reasonably participate (e.g. staggering, slurred speech) were not approached or invited to participate. Participants were asked about both their current drunkenness and their expected drunkenness in order to include people who would otherwise be too drunk to be interviewed later in the night. Compliance rates for those asked to participate were 50.6% in City 1, 48.7% in City 2 and 57.2% in City 3. A total of 467 individuals completed the questionnaire, of whom 454 had already consumed, or intended to consume, alcohol that night. For the purpose of this study, we focused on alcohol consumers aged 18–35 years (*n* = 416). Eight participants that had not provided values for all scales of interest were excluded from analyses resulting in a final sample of 408. Data were analysed in SPSS (v21) using descriptive statistics, chi squared and logistic regression (enter method).

Ethical approval for the study was obtained from Liverpool John Moores University’s Research Ethics Panel.

## Results

Over half (55.6%) of participants were male (Table 1) and median age was 22 years. City samples varied by gender (more males in city 2, 65.6%, *P* = 0.024). Most (96.3%) participants had consumed alcohol at the point of survey (median of 10 UK units) and 64.0% reported having preloaded. Median scale values for all cities combined were: personal drunkenness at survey, 4; expected personal drunkenness, 7; perceived city drunkenness norm, 8; and ideal personal drunkenness, 7. Females tended to rate perceived city drunkenness norm as higher and their ideal personal drunkenness as lower than males. Rating perceived city drunkenness norm as high was also less common among participants from City 2, likely reflecting the greater proportion of males surveyed in this location. There were no differences across age categories. However, preloading was associated with higher ratings across all scales (Table 1).

**Table 1:** Sample characteristics, median scale values and percentage exceeding median scale values

Sample	%	n	Personal drunkenness at survey	Expected personal drunkenness	Perceived city drunkenness norm	Ideal personal drunkenness
Median value			4	7	8	7
% exceeding median scale value						
All	100	408	41.2	40.9	48.5	23.0
Gender						
Male	55.6	227	40.1	44.1	37.4	26.9
Female	44.4	181	42.5	37.0	62.4	18.2
*X*^*2*^			0.250	2.062	25.169	4.240
P			0.617	0.151	< 0.001	0.039
Age group (years)						
18–20	33.3	136	41.9	42.6	46.3	27.2
21–24	34.6	141	39.7	45.4	53.9	23.4
25–35	32.1	131	42.0	34.4	45.0	18.3
*X*^*2*^			0.190	3.671	2.533	2.987
P			0.909	0.160	0.282	0.225
Location						
City 1	46.8	191	40.8	40.8	55.5	17.8
City 2	30.6	125	41.6	36.0	33.6	26.4
City 3	22.5	92	41.3	47.8	54.3	29.3
*X*^*2*^			0.019	3.067	16.114	5.817
P			0.991	0.216	< 0.001	0.055
Preloaded						
No	36.0	147	32.7	32.0	40.1	17.0
Yes	64.0	261	46.0	46.0	53.3	26.4
*X*^*2*^			6.892	7.628	6.481	4.716
P			0.009	0.006	0.011	0.030

**Fig. 1:**
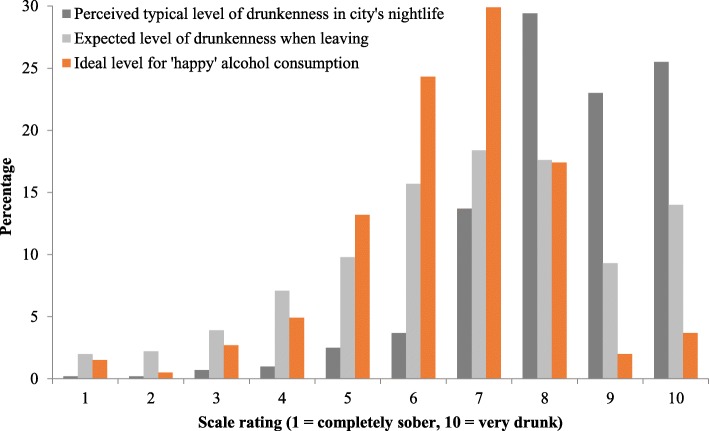
Distribution of expected personal drunkenness, perceived city drunkenness norms and ideal personal level of drunkenness

**Fig. 2:**
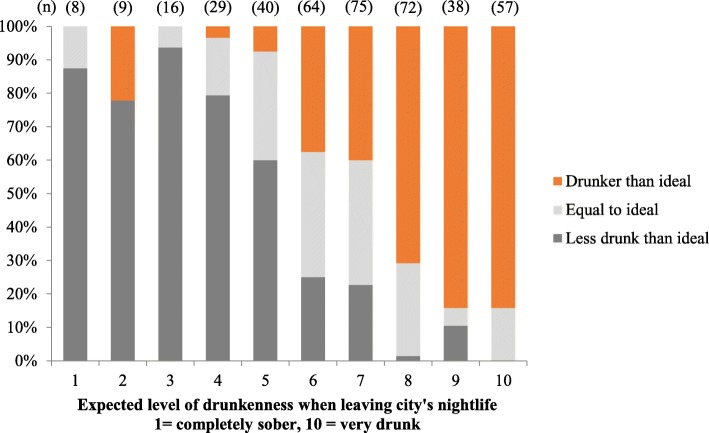
Proportion expected to be drunker, equal or less drunk than their ideal when leaving nightlife

Figure 1 shows the distribution of scale values for expected personal drunkenness, perceived city drunkenness norms and ideal level of drunkenness. Over three quarters (77.9%) of participants rated typical drunkenness of people in the nightlife area they were visiting at 8 or above although only 40.9% expected to reach this level of drunkenness themselves and only 23.1% reported an ideal level of drunkenness of 8 or more. Thus, over three quarters (76.2%) of individuals reported that their ideal level of intoxication was below what they perceived to be the norm in the city they were visiting (14.5% equal, 9.3% higher) and almost half (46.8%) expected to leave the city drunker than their ideal personal drunkenness (25.2% at their ideal and 27.9% below it). Figure 2 shows the proportion of participants expecting to have exceeded, equalled or be below their ideal level of drunkenness when leaving nightlife based on their expected level of drunkenness. The vast majority (78.4%) of those expecting to reach 8 or above on the scale were expecting to exceed their ideal level of drunkenness.

In logistic regression analysis (see Table 2), high expected drunkenness (above the median, ≥8) was associated with higher ideal level of drunkenness, higher perception of typical nightlife drunkenness and later expected home time, as well as earlier survey time and higher-self reported drunkenness at survey. There were no relationships with demographics, preloading or quantity of alcohol consumed by point of survey. In a separate model, high ideal level of personal drunkenness (above the median, ≥8) was associated with earlier survey time and higher self-rated drunkenness at survey, with the relationship with later home time just failing to reach significance (Table 2).

**Table 2:** Logistic regression analysis of factors associated with high expected or high ideal drunkenness

		High expected drunkenness (above median, ≥8)				High ideal drunkenness (above median, ≥8)			
		95% CIs				95% CIs			
		AOR	Lower	Upper	P	AOR	Lower	Upper	P
Ideal drunkenness		1.42	1.14	1.76	0.002	–	–	–	–
Perceived norm drunkenness		1.28	1.04	1.59	0.023	1.00	0.83	1.21	0.976
Survey time *(hour)*		0.44	0.35	0.55	< 0.001	0.72	0.59	0.88	< 0.001
Drunkenness at survey		1.88	1.54	2.28	< 0.001	1.55	1.31	1.82	0.001
Alcohol units by survey		1.01	0.98	1.05	0.453	1.01	0.98	1.04	0.440
Expected home time *(hour)*		2.07	1.66	2.57	< 0.001	1.17	0.99	1.39	0.068
Gender (ref: Male)	Female	1.17	0.62	2.20	0.628	0.71	0.39	1.27	0.246
City (ref: City 1)	City 2	0.68	0.34	1.36	0.276	1.48	0.78	2.82	0.235
	City 3	1.07	0.53	2.17	0.857	1.51	0.76	2.97	0.236
Preloaded (ref: no)	Yes	0.70	0.38	1.29	0.254	1.10	0.60	2.03	0.758
Age group (ref: [18–20])	21–24	1.44	0.74	2.81	0.287	0.90	0.48	1.68	0.731
	25–35	0.78	0.38	1.62	0.505	0.56	0.28	1.12	0.103

*n* = 394; 14 cases were excluded due to missing data on alcohol consumption, survey or expected home time; ref. = reference category; 95% CIs = 95% Confidence Intervals; *AOR* adjusted odds ratio

## Discussion

Our study provides unique quantitative data indicating two important features of nightlife drinking behaviours. Firstly, a substantial proportion of nightlife patrons expected to become drunker whilst on a night out than their personal ideal level of drunkenness where they are as happy as they can be after drinking alcohol. Secondly, nightlife users’ perceptions of typical drunkenness levels in the nightlife environment they were visiting appeared to be elevated. Both of these findings may support emerging work to address cultures of intoxication in nightlife environments.

Almost half of participants were expecting to drink beyond their ideal level of happy drunkenness on the night of survey and this increased to four fifths of those who intended to reach the highest levels of intoxication (i.e. ratings of 8 and above; Fig. 2). In some respects, this reflects findings from qualitative research exploring cultures of intoxication among young people, where extreme drinking has been described using terms such as ‘calculated hedonism’ [21] and ‘determined drunkenness’ [31]; a planned behaviour under the control of the drinker. However, it may also suggest that many drinkers that expect to become extremely drunk may in fact prefer not to reach such a high level of intoxication, but rather are driven by social or other pressures to conform. Drinkers have been found to seek a ‘diminished but not eradicated’ sense of self-responsibility [32, 33] and to dislike the loss of control experienced when becoming too drunk and experiencing associated negative effects (e.g. dizziness, vomiting). Young drinkers often assess their level of drunkenness based on bodily signs of intoxication [32] and report implementing strategies in response to such signs in attempts to control intoxication, such as changing drink types, slowing down consumption and stopping drinking all together [21]. However, by the time signs indicative of extreme drunkenness are observed, further alcohol may already in the system that has yet to be absorbed. Thus the point at which people realise they have had overconsumed alcohol can be too late to avert negative effects.

Alcohol messages promoting ‘responsible’ drinking in nightlife settings have typically focused on the negative aspects of extreme drunkenness (e.g. vomiting, injury, assault), with limited evidence of effectiveness [12]. Such proscriptive messages have been criticised for ignoring the fun and desirable aspects of drunkenness, and potentially being counter-productive by reinforcing cultures of intoxication [12]. Research examining reactions to alcohol campaigns has suggested that positively framed messages evoking feelings of happiness may be more successful than those focusing on negative outcomes [34]. Thus alcohol messages in nightlife may be better targeted at encouraging drinkers to recognise and react to signs that they are reaching their ideal ‘happy’ level of drunkenness rather than the consequences of having moved beyond this. This may be controversial for public health bodies seeking to prevent alcohol-related harm, yet combined with broader work to affect social norms and tolerance of drunkenness it may help shift all aspects of drunkenness measured here down the scale.

Social norms research shows that young people often overestimate alcohol use by their peers, and that perceived alcohol norms are related to personal alcohol use [26]. Here, four fifths of individuals rated typical level of nightlife drunkenness at 8 or above yet only two fifths expected to reach this level themselves. Perceiving typical nightlife drunkenness as ≥8 was associated with higher expected alcohol consumption. Whilst further research is needed to tease out the effects of perceived drunkenness norms on individual behaviour, our study suggests that interventions which seek to correct misperceptions of normal levels of drunkenness in nightlife environments may be of benefit. Evidence for the effectiveness of social norms interventions in reducing alcohol use is largely limited to their use among university and college students. Here, a review of 70 studies found some significant impacts on drinking behaviours and alcohol problems, yet concluded that effect sizes were too small to be of relevance to policy and practice [35]. However, most studies had focused on the provision of social norms information to individual drinkers with few having used a settings approach to explore the impact of social marketing campaigns across campuses. Studies of social marketing campaigns for alcohol use in campus settings report mixed results [35, 36], and very little is known about the applicability of such campaigns beyond educational settings, including in nightlife environments. Thus examining the potential of such campaigns to change perceptions of drinking norms and modify drinking behaviour among nightlife patrons is an important gap for future research.

Finally, expected levels of drunkenness were associated with later expected home time. This reflects previous research suggesting that late night drinking environments are dominated by heavy drinking individuals [4–7]. Extended licensing hours were introduced in the UK in 2005 in an attempt to promote a more relaxed late night drinking culture. However, increasing access to alcohol is unlikely to function as a remedy for alcohol-related harm in a culture of intoxication. Reversal of such an approach is now occurring in parts of Australia where in response to high levels of alcohol-related harm, alcohol service hours have been reduced in several cities. Evaluations are showing substantial reductions in alcohol-related problems associated with such restrictions [16, 17, 37, 38]. With no such policy reversal imminent in the UK, local authorities are turning their attention to community-based approaches that aim to increase compliance with legislation preventing the sales of alcohol to drunks and alter the social norms that make nightlife drunkenness acceptable [39, 40]. Our study provides initial data suggesting that extreme drunkenness is less desirable and ubiquitous than currently perceived.

This study faced a number of limitations common to nightlife surveys. Data were collected opportunistically, sample sizes in each city were relatively small and overall compliance was 51%, thus samples cannot be considered representative of all nightlife users in the three cities. Further, individuals who were visually assessed as being severely intoxicated were excluded to meet ethical requirements [41]. However, conducting surveys across several hours and enquiring about expected drunkenness at the end of the night permitted the inclusion of individuals who may later have been excluded due to extreme intoxication. We did not ask participants the reason for their night out or if their alcohol consumption on the night of survey was typical of their usual nightlife drinking behaviour and it is therefore possible that individuals were on either more relaxed or heavier drinking sessions. We also did not measure participants’ blood alcohol concentration or record visible signs of intoxication to assess against perceived levels of intoxication. These types of measures would be useful for inclusion in future studies.

## Conclusions

Excessive drinking and drunkenness in nightlife environments damages public health, drains public resources and poses an ongoing challenge to public services. Addressing cultures of intoxication in nightlife environments requires a better understanding of the social norms that support them. Our study suggests that UK nightlife patrons may have elevated perceptions of typical nightlife drunkenness levels and that substantial proportions may drink beyond their ideal level of drunkenness. Findings could support social marketing approaches to correct social perceptions of drunkenness and can inform further work both in the UK and internationally to better understand the drivers of and potential solutions to harmful drinking behaviours in nightlife environments.

## Additional file

Additional file 1: Questionnaire content. Questions used in the study. (PDF 244 kb)

## Abbreviations

AOR: Adjusted odds ratio
CI: Confidence interval
Ref: Reference category
