# Supplementary material for: Ideal, expected and perceived descriptive norm drunkenness in UK nightlife environments: a cross-sectional study
Source: BMC Public Health. 2019 Apr 27;19:442. doi: 10.1186/s12889-019-6802-5 (PMC6486975; doi:10.1186/s12889-019-6802-5)
Supplement: Supplementary file 1 — Questionnaire content. Questions used in the study. (PDF 244 kb) [file 12889_2019_6802_MOESM1_ESM.pdf]

Questionnaire content – questions used in the study

Location		Date		Time	

**What is your age?**	

How would you describe your gender?

Male		Female		Transgender	

**Are you a student?**	Yes		No	

**What time did you come into the city centre for your night out?**	

Have you consumed any alcohol tonight?

Yes		No	

**If yes, what time did you have your first alcoholic drink?**	

Did you consume any alcohol before coming out, for example at home, a friend's home or a hotel room?

Yes		No	

If yes, can you tell me what you drank?
(Researcher notes drink quantity and size)

Beer or lager	
Cider	
Wine	
Alcopops	
Shots of spirits	

After leaving the *home/hotel room* did you consume any alcohol elsewhere before coming into the city centre's nightlife? (e.g. local pub, public transport, restaurant?

Yes, where		No	

If yes, can you tell me what you drank?

Beer or lager	
Cider	
Wine	
Alcopops	
Shots of spirits	

Since you have been in the city centre's nightlife what alcohol have you drank that was bought in bars/pubs/clubs ?

Beer or lager	
Cider	
Wine	
Alcopops	
Shots of spirits	

And what alcohol have you drank that was bought from off-licensed premises or supermarkets, including any alcohol you might have brought out with you?

Beer or lager	
Cider	
Wine	
Alcopops	
Shots of spirits	

**What time do you plan on leaving the city centre's nightlife tonight?**	

On a scale of 1 to 10, with 1 completely sober and 10 very drunk, please answer the following questions:

How drunk do you feel at the moment?

1	2	3	4	5	6	7	8	9	10

How drunk do you think you will be when you leave the city centre's nightlife tonight?

1	2	3	4	5	6	7	8	9	10

Overall, what do you think is the typical level of drunkenness that people reach on a night out in the city centre?

1	2	3	4	5	6	7	8	9	10

For you personally, what do you think is the ideal level where you are as happy as you can be after drinking alcohol?

1	2	3	4	5	6	7	8	9	10
